# Inhibiting the Cholesterol Storage Enzyme ACAT1/SOAT1 in Aging Apolipoprotein E4 Mice Alters Their Brains’ Inflammatory Profiles

**DOI:** 10.3390/ijms252413690

**Published:** 2024-12-21

**Authors:** Thao N. Huynh, Emma N. Fikse, Adrianna L. De La Torre, Matthew C. Havrda, Catherine C. Y. Chang, Ta Yuan Chang

**Affiliations:** 1 Department of Biochemistry and Cell Biology, Geisel School of Medicine at Dartmouth, Hanover, NH 03755, USA; thao.n.huynh.gr@dartmouth.edu (T.N.H.);; 2 Department of Molecular and System Biology, Geisel School of Medicine at Dartmouth, Hanover, NH 03755, USA

**Keywords:** apolipoprotein E4 (*APOE4*), microglia, cholesterol, cholesteryl esters, acyl-CoA:cholesterol acyltransferase, sterol O-acyltransferase 1, ACAT inhibitor, ATP binding cassette subfamily A member 1, Alzheimer’s disease, F12511, LOAD, late-onset Alzheimer’s disease, TLR4, NFκB, lipid rafts, DSPE-PEG_2000_, phosphatidylcholine, interleukin-1 beta

## Abstract

Aging and apolipoprotein E4 (*APOE4*) are the two most significant risk factors for late-onset Alzheimer’s disease (LOAD). Compared to *APOE3*, *APOE4* disrupts cholesterol homeostasis, increases cholesteryl esters (CEs), and exacerbates neuroinflammation in brain cells, including microglia. Targeting CEs and neuroinflammation could be a novel strategy to ameliorate *APOE4*-dependent phenotypes. Toll-like receptor 4 (TLR4) is a key macromolecule in inflammation, and its regulation is associated with the cholesterol content of lipid rafts in cell membranes. We previously demonstrated that in normal microglia expressing APOE3, inhibiting the cholesterol storage enzyme acyl-CoA:cholesterol acyltransferase 1 (ACAT1/SOAT1) reduces CEs, dampened neuroinflammation via modulating the fate of TLR4. We also showed that treating myelin debris-loaded normal microglia with ACAT inhibitor F12511 reduced cellular CEs and activated ABC transporter 1 (ABCA1) for cholesterol efflux. This study found that treating primary microglia expressing *APOE4* with F12511 also reduces CEs, activates ABCA1, and dampens LPS-dependent NFκB activation. In vivo, two-week injections of nanoparticle F12511, which consists of DSPE-PEG_2000_, phosphatidylcholine, and F12511, to aged female *APOE4* mice reduced TLR4 protein content and decreased proinflammatory cytokines, including IL-1β in mice brains. Overall, our work suggests nanoparticle F12511 is a novel agent to ameliorate LOAD.

## 1. Introduction

Alzheimer’s disease (AD) is a progressive form of dementia that significantly reduces patients’ quality of life. Those with AD experience memory loss, cognitive decline, and behavioral changes. Aging is the leading cause of AD and is associated with late disease onset (LOAD) [1]. About 90–95% of AD patients have LOAD [2]. Currently, 1 in 9 people aged 65 years and older have the disease [3]. As people age, the risk of having AD increases significantly. The *APOE4* gene variants further elevate this risk in carriers by 4 to 15-fold [4,5]. It is not clear how *APOE4* increases the risk for AD. APOE is a protein that primarily functions in cholesterol and lipid handling in the body and the brain [5]. APOE has three different isoforms (E2, E3, and E4), in which E2 is associated with lower disease risk, E3 is recognized as associated with the “normal” phenotypes, and E4 is associated with increased AD risk [5]. Interestingly, between these three isoforms, there are only two amino acid differences in their sequence, which drastically impact their downstream phenotypes [5]. *APOE4* expression is reported to cause dysfunction across multiple cell types in the central nervous system (CNS), the periphery, and the vasculature [6,7,8,9,10,11,12].

In 1907, when Dr Alzheimer described the pathological features of his original patient, he focused on amyloid plaques and neurofibrillary tangles in the brain [13]. However, shortly afterward (from 1909 to 1911), Dr. Alzheimer and his colleague published several papers reporting that the original patient’s brain contained lipid-rich materials in glial cells, the vasculature, and the ganglion. These works were written in German but were reviewed in English by Foley in 2010 [14]. Thus. Dr Alzheimer’s subsequent works indicate that lipid deposit in the brain is the third pathological feature in Alzheimer’s patients (Reviewed in [14,15,16]). More recent data from Alzheimer’s disease patients’ brains suggested aberrant lipid metabolism [9,17]. These pathological hallmarks in AD have been demonstrated to be associated with neuroinflammation, which is correlated with worsened disease progression [18]. Much AD research focuses on tackling Aβ plaques and NFT to ameliorate disease progression and symptoms, with less focus on lipids [17]. Recently, more advanced techniques have allowed researchers to further investigate the role of lipids and cholesterol in disease pathogenesis and progression (Reviewed in [19]).

In recent years, progress towards developing AD therapies has been made, with the FDA approving a few amyloid-targeted immunotherapy. However, recent studies have shown that some patients carrying two copies of the *APOE4* gene had died from amyloid-related imaging abnormalities (ARIA). Clinical studies suggest that some AD patients with *APOE4* homozygotes may be more prone to experience serious side effects from ARIA in response to amyloid immunotherapy [20,21]. *APOE4* is speculated to increase ARIA risk in patients due to its ability to damage the blood-brain barrier integrity, elevate neuroinflammation, and increase cerebral amyloid angiopathy (CAA) [21]. Thus, there is an urgent need for alternative therapies for the *APOE4* patient population.

In the brain, APOE4 protein is produced mainly by two cell types: astrocytes and microglia. APOE4 protein impairs immune responses and increases lipid droplet accumulation in the microglia [10,11,12] (Reviewed in [19,22]). Increased lipid droplet accumulation in *APOE4* microglia is associated with AD pathology and worsened neuroinflammation [10,11,12] (Reviewed in [19,22]).

Lipid droplets contain neutral lipids, predominantly triacylglycerol (TAG) and cholesteryl ester (CE). While most recently published studies focused on removing excess TAG to improve *APOE4* phenotypes [22], studies on removing excess CE are limited. In cells, CE is synthesized by the cholesterol storage enzyme acyl-CoA:cholesterol acyltransferase, also known as sterol O-acyltransferase 1 (ACAT1/SOAT1). Genetic ablation and pharmaceutical inhibition of ACAT1/SOAT1 have been reported to reduce CE, ameliorate AD-associated pathological hallmarks, and attenuate neuroinflammation via modulating toll-like receptor 4 (TLR4) [23,24,25,26,27,28,29,30]. In the context of *APOE4*, pharmaceutical inhibition of ACAT/SOAT using Avasimibe (CI-1011) was reported to be beneficial in 5xFAD/*APOE4* mice by reducing intracellular lipid droplets and neuroinflammation, improving memory performance, as well as restoring postsynaptic protein levels [29]. However, whether ACAT1/SOAT1 pharmaceutical inhibition is beneficial in an *APOE4* aging model, which captures two of the most significant risk factors for AD, remained unknown. In this manuscript, we investigate the effect of ACAT1/SOAT1 inhibition in vitro (*APOE4* microglia) and in vivo (aging *APOE4* mice). Below, we report our findings in detail.

## 2. Results

### 2.1. F12511 Treatment in Primary APOE4 Microglia Reduces Lipid Droplets and Increases ABCA1 Protein Content

*APOE4*-expressing microglia are reported to have neutral lipid droplet accumulation and secrete higher levels of proinflammatory cytokines [7,10,11,12,31]. We previously showed that ACAT1 inhibitors reduce lipid droplets and produce anti-inflammatory responses in microglia and macrophages [28,32,33,34]. Here, we chose to study ACAT1 inhibitors in *APOE4* primary microglia. To establish an in vitro system, we first isolated primary microglia from *APOE3* and *APOE4* target replacement mice (*APOE3-* and *APOE4-TR*) following the procedure described in Section 4 and in Figure S1A. We then exposed these microglia to the ACAT inhibitor F12511, as shown in Figure S1B. We chose to use F12511 as the ACAT inhibitor for this study, as our laboratory had previously developed a drug delivery system using F12511 for ACAT1 inhibition in vivo [30]. Primary microglia were allowed to rest in serum-free media overnight, and all subsequent treatment was performed for less than 24 h and in serum-free condition to ensure primary microglia transcriptome is more native-microglia-like [35].

**Figure 1:**
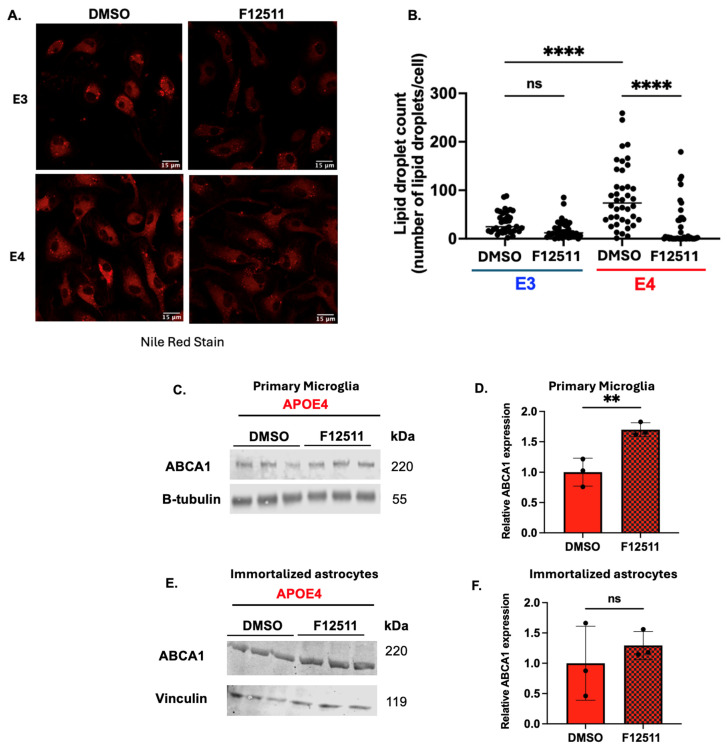
In vitro F12511 treatment reduces lipid droplets and upregulates ABCA1 protein content in primary *APOE4* microglia but not in immortalized *APOE4* astrocytes. (**A**) Nile Red assay in primary *APOE3* and *APOE4* microglia treated with or without F12511. Scale bar: 15 μm. (**B**) Lipid droplet quantification from Nile Red data. N = 40 cells per treatment group were analyzed. The procedure for Nile red assay and imaging analysis were described in Section 4. (**C**) Representative Western blot monitoring ABCA1 protein content in *APOE4* primary microglia treated with or without F12511. (**D**) Quantification of Western blot data. The procedures for Western blot analysis and quantitation are described in Section 4. (**E**) Representative Western blot monitoring ABCA1 protein content in *APOE4* immortalized astrocytes treated with or without F12511. (**F**) Quantification of Western blot data. N = 3 for Western blot experiments. The value of cells treated with DMSO was normalized to 1. Data are expressed as mean ± SEM. ** *p* < 0.01; **** *p* < 0.0001; ns: not significant.

We first tested whether ACAT1 inhibition would reduce lipid droplets in *APOE4* primary microglia by treating cells with F12511 for 12 h and staining them with Nile Red, a dye that is widely used for visualizing intracellular lipid droplets (containing mainly CE and TAG) [36]. Nile Red has been used to monitor the effect of ACAT1 inhibition [32,37,38]. Nile Red-stained cell images and quantification results are shown in Figure 1A,B. These results showed that *APOE4* microglia exhibited a higher lipid droplet count than the value found in *APOE3* primary microglia, suggesting *APOE4* primary microglia has more neutral lipid droplets at baseline. This result agrees with previous studies from Machlovi et al. and Litvinchuk et al. [10,11]. Treatment with ACAT1/SOAT1 inhibitor F12511 did not significantly reduce lipid droplet count in *APOE3* primary microglia, as their lipid droplet level is already low, while in *APOE4* primary microglia, F12511 treatment significantly reduced lipid droplets count. These results indicated that besides higher levels of TAG as previously reported by other research groups in literature, there is also a higher level of CE in *APOE4* primary microglia when compared to its *APOE3* counterpart, and this CE-rich lipid droplet pool in *APOE4* primary microglia is sensitive to ACAT1/SOAT1 inhibition.

We next asked what the consequence of CE lipid droplet reduction would be when ACAT1/SOAT1 is inhibited in *APOE4* primary microglia. Previously, we had demonstrated that in human microglia HMC3 cells loaded with cholesterol-rich myelin debris, ACAT1/SOAT1 inhibition reduced CE accumulation and increased cholesterol efflux via upregulation of ATP transporter cassettes A1 (ABCA1) gene expression and protein content [32]. Since APOE4 primary microglia was reported to have disrupted cholesterol metabolism and increased lipid droplet accumulation [8,10,11], we speculated that ACAT1 inhibition might also upregulate ABCA1 content in *APOE4* primary microglia. To test, we treated the *APOE4* primary microglia with F12511 for 12 h, followed by monitoring ABCA1 protein content on Western blot and quantified ABCA1 signal. (Figure 1C,D) Our results reveal that ABCA1 protein content did increase with F12511 treatment, suggesting that ACAT1 inhibition might also alleviate the cholesterol burden in *APOE4* primary microglia cells by activating the ABCA1 transporter [32].

As different cell types in the CNS play different roles in cholesterol metabolism, the effect of F12511 is likely cell-type dependent. Astrocytes are believed to be responsible for most cholesterol synthesis in the CNS. In *APOE4*-expressing human iPSC-derived astrocytes, cholesterol accumulates in the lysosome [8,39]. To test if F12511 can also increase ABCA1 protein in astrocytes, we treated immortalized *APOE4* astrocytes [40] with F12511 and monitored their ABCA1 protein content on Western blot (Figure 1E,F). The result showed that unlike in microglia, F12511 treatment in the *APOE4* astrocyte cell line did not impact ABCA1 protein content. A previous study showed that in *APOE4* immortalized astrocytes, the ABCA1 protein level was lower than its *APOE3* counterpart [41]. However, the authors also reported that the cellular ABCA1 protein expression level in *APOE4* primary astrocytes is comparable to that of *APOE3* astrocytes [41]. Our current result, shown in Figure 1, suggests that the effect of F12511 on *APOE4* microglia may be cell-type dependent; however, the impact of ACAT1 inhibition in primary *APOE4* astrocytes needs to be further examined.

### 2.2. Pharmacological Inhibition of ACAT1 by F12511 Dampen NFκB Activation in Primary E4 Microglia in a TLR4 Dependent Manner

Next, we focused on understanding the biological consequence of reducing CE-rich lipid droplets through ACAT inhibition in *APOE4* microglia. Evidence suggests that *APOE4* carries out damaging AD-related phenotypes in a TLR4-dependent pathway [42,43,44]: LPS brain infusion in *APOE4* mice results in increased NFκB activation and IL-1β secretion compared to *APOE3* counterpart [43,44]. We thus chose to evaluate the response of *APOE4* microglia to an inflammation stimulus using lipopolysaccharide (LPS). LPS is an inflammation agent; it is a TLR4-specific ligand in microglia and is commonly used for studying TLR4 and its signaling pathway.

We had previously shown that in LPS-treated microglial N9 cells, ACAT1 inhibition promotes TLR4 endocytosis from the plasma membrane to the endo/lysosomal compartment for degradation; ACAT1 inhibition acts by dampening NFκB activation and proinflammatory responses via modulating the fate of TLR4 [28]. TLR4 is located at the plasma membrane lipid raft domain, which is heterogeneous in function and composition; those that are cholesterol-rich can serve as platforms to facilitate various receptor signaling events. Altering cholesterol content within the lipid raft microdomain can regulate membrane fluidity and directly affect immune receptor signaling, such as TLR4 (Reviewed in [45,46]). Our previous results in microglia N9 cells suggested that ACAT1 blockage may regulate cholesterol content in the TLR4-containing lipid raft microdomain [28]. We thus speculated that ACAT1 inhibition might also provide similar benefits to *APOE4* microglia. To test this possibility, we monitored NFκB activation in LPS-treated *APOE4* microglia with or without F12511 treatment. Cells without LPS exposure were used as controls.

**Figure 2:**
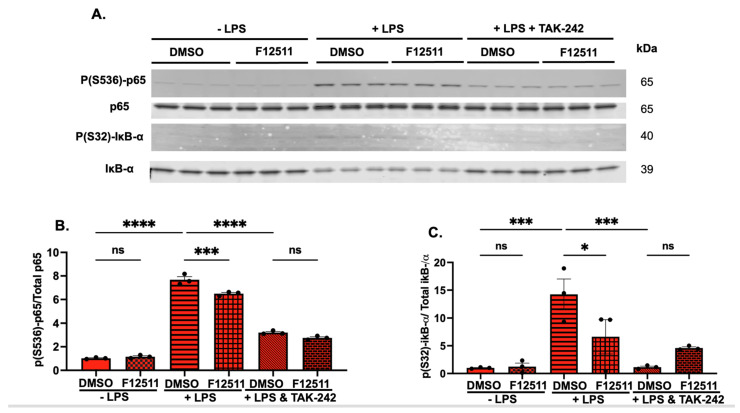
Pharmaceutical inhibition of ACAT1 by F12511 dampens NFκB activation in LPS-induced primary E4 microglia in a TLR4-dependent (TAK-242 sensitive) manner. (**A**) Representative Western blot of keys NFκB activation markers P(S536)-p65, p65, P(S32)-IκB-α, IκB-α. Western blot quantification for (**B**) P(S536)-p65/p65 ratio and (**C**) P(S32)-IκB-α/IκB-α ratio. N = 3. The value of cells treated with DMSO with no LPS was normalized to 1. Data are expressed as mean ± SEM. * *p* < 0.05, *** *p* < 0.001, **** *p* < 0.0001.

To validate whether the effect of F12511 is TLR4-dependent, we treated cells exposed to LPS with or without F12511 with a TLR4-specific inhibitor—TAK242 [47]. The treatment scheme and experimental setup are graphically illustrated in Figure S2. The phosphorylation state of p65 and iκβ-α were monitored to evaluate the NFκB activation state [48]. A representative Western blot is shown in Figure 2A, and the quantification of these two markers (p65 and Iκβ-α) is reported in Figure 2B (p65) and Figure 2C (iκβ-α), respectively. Our analysis revealed that F12511 treatment in *APOE4* microglia dampens NFκB activation when both markers were examined (third and fourth bars, Figure 2B,C). Additional experiments showed that treatment of these cells with the TLR4-specific inhibitor TAK242 abolished the effect of F12511 in *APOE4* cells exposed to LPS, supporting the conclusion that the impact of F12511 on dampening NFκB activation is dependent on TLR4 signaling (fifth and sixth bar, Figure 2B,C) Overall, our result demonstrated that treatment of the ACAT1 inhibitor F12511 in *APOE4* primary microglia cells dampens NFκB activation in a TLR4-dependent manner. This result agrees with our previously published data in N9 cells carrying mouse *apoe* and using a different ACAT inhibitor, K604 [28]. Together, these datasets suggest that the mechanism responsible for ACAT1 blockade dampening NFκB through the TLR4 pathway is conserved regardless of APOE isoform in both humans and mice.

### 2.3. Design of F12511 In Vivo Efficacy Studies in APOE3 and APOE4 at Different Age Ranges

**Figure 3:**
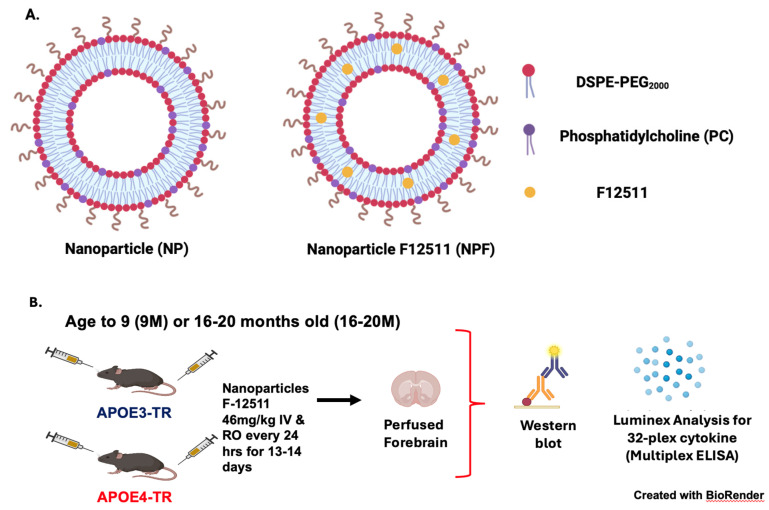
Design of F12511 in vivo efficacy studies in *APOE3* and *APOE4* KI mice at two different age ranges. (**A**) Diagram and components of nanoparticle with or without ACAT1 inhibitor F12511. Nanoparticles comprise DSPE-PEG2000 and PC, according to the procedure published in [49]. (**B**) Animal treatment scheme. Mice were aged 9 months old (9 M) or 16–20 months old (16–20 M) for this study, followed by daily injection by alternate intravenous (IV) and retro-orbital (RO) routes. Mice were then perfused with PBS, and forebrain tissues were collected and homogenized for Western blot and Luminex analysis. See Section 4 for details. Created with BioRender.

Next, we aim to test the efficacy of ACAT1 inhibitors in vivo. We employed a lipid-based nanoparticle system encapsulating the ACAT1/SOAT1 inhibitor F12511 that was previously published and tested in wild-type (WT) and triple transgenic (3xTg) AD mice [30,49]. A graphical diagram describing this system is illustrated in Figure 3A,B.

Employing our nanoparticle system, we injected adult (9 M-old) and aged (16–20 M-old) female *APOE3-TR* and *APOE4-TR* mice with PBS (control), nanoparticle (vehicle), and nanoparticle F12511 daily for 13–14 days. We chose to use exclusively female mice in this study, as other studies have reported that female *APOE4* mice tend to experience worsened neuroinflammation compared to their male counterparts [50,51]. We collected forebrain tissues and analyzed key inflammatory markers using Luminex xMAP technology and Western blot. As AD is an aging-related disease, we designed our studies to examine the effect of nanoparticle F12511 in *APOE4-TR* adult and aged mice to determine its efficacy at two different ages.

### 2.4. Two Weeks of Daily IV Injection of Nanoparticle F12511 at 46 mg/kg and PC Subtly Changes the Inflammatory Profile in the Forebrains of 9 M-old APOE4 Mice, but Not APOE3 Mice

We first examined the effect of nanoparticles with PC and F12511 (NPF) in 9 M-old adult *APOE3* and *APOE4* mice. Since we saw that acute 12 h treatment with F12511 was able to dampen NFκB activation in a TLR4-dependent manner (Figure 2) and treatment with another ACAT1/SOAT1 inhibitor, K604 was able to reduce TLR4 protein content in N9 microglia after 48 h treatment [28], we speculated that with two weeks of daily treatment, NPF might decrease TLR4 protein content in the brain.

**Figure 4:**
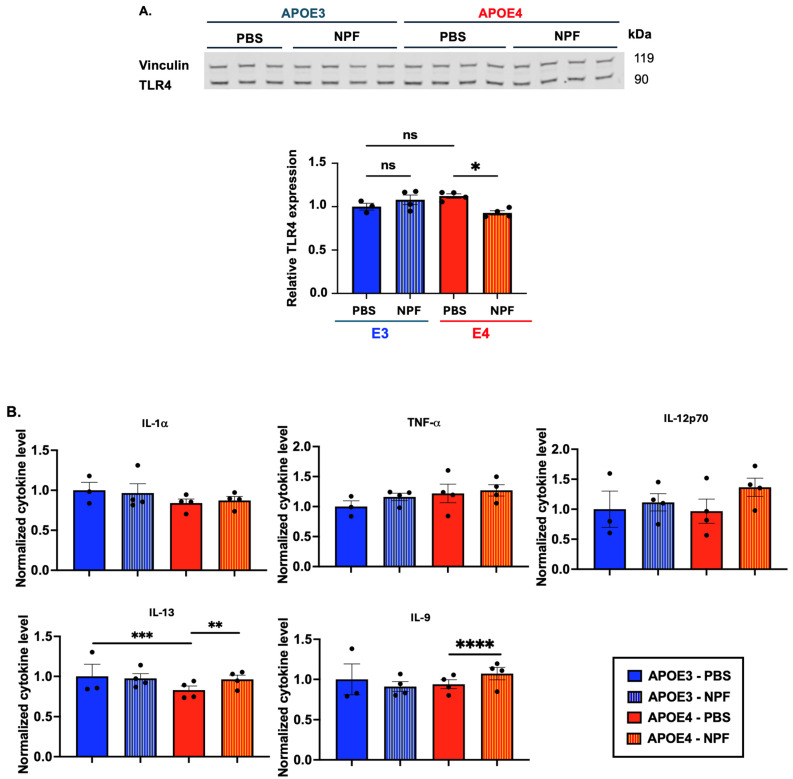
Two weeks of daily alternate IV and RO injections of NPF at 46 mg/kg subtly change the inflammatory profile in 9 M-old *APOE4* mice but not in APOE3 mice. (**A**) Representative Western blot and quantitative analysis of relative TLR4 expression in 9 M-old mice forebrain homogenate. Quantitative analysis revealed that NPF slightly reduced total TLR4 protein expression in *APOE4* forebrain homogenate but not in *APOE3* mice. (**B**) Detectable Alzheimer’s-related cytokines and cytokines significantly altered by drug treatment from MILLIPLEX xMAP analysis were plotted individually. IL-13 and IL-9 changed significantly in *APOE4*-treated forebrain homogenates, while APOE3 mice were unaffected. The value of *APOE3* mice treated with PBS injection was normalized to 1. Data are expressed as mean ± SEM. * *p* < 0.05,** *p* < 0.01, *** *p* < 0.001, **** *p* < 0.0001.

After treatment with NPF, we observed a slight decrease in TLR4 protein content in the 9 M-old *APOE4* mice forebrain but not in their *APOE3* counterpart (Figure 4A). To determine whether this drop in TLR4 protein content in *APOE4* mice correlates with a pro-inflammatory response in the forebrain region, we analyzed the same tissues using Miliplex xMAP technology to assess different pro- and anti-inflammatory cytokines levels. The analyzed cytokines within the detectable range are reported in Figure 4B. Proinflammatory cytokines such as IL-1α, TNFα, and IL-12p70 downstream of NFκB activation did not change significantly. Interestingly, NPF treatment in *APOE4* mice slightly increases cytokines such as IL-9 and IL-13 compared to PBS-treated mice. The role of IL-9 and IL-13 in *APOE4* mice is unknown. However, IL-13 is generally recognized as an anti-inflammatory cytokine. In an amyloid precursor protein (APP) mouse model for AD—APP23, intracerebral injection of IL-4/IL-13 increases Aβ clearance and improves cognitive deficits [52]. Additionally, it is unknown how IL-13 and TLR4 signaling correlate in microglia, but in epithelial cells, IL-13 decreases TLR4 function [53].

In adult mice, we observed a subtle effect from NPF treatment in *APOE4* mice but not in *APOE3* mice. This difference might be due to *APOE4* mice exhibiting a leaky BBB as part of their disease phenotype [54], which allows more nanoparticles to enter the brain cells and alter the brain’s inflammatory profile. We next examined the effect of nanoparticle F12511 treatment in aging *APOE3* and *APOE4* mice, where the BBB is leaky as aging progresses.

### 2.5. Two Weeks of Daily Alternate IV Injection of NPF at 46 mg/kg Changes PLIN2, TLR4 Protein Content, and Inflammatory Profile in the Forebrains of 16–20 M-old APOE3 and APOE4 Mice

As the *APOE4* phenotype exacerbates with age, we aged *APOE3* and *APOE4* mice to 16–20 M-old to test the effect of NPF. We had previously observed in 3xTg AD mice that NPF displayed prominent effects in the 16–20 M-old age group [30].

**Figure 5:**
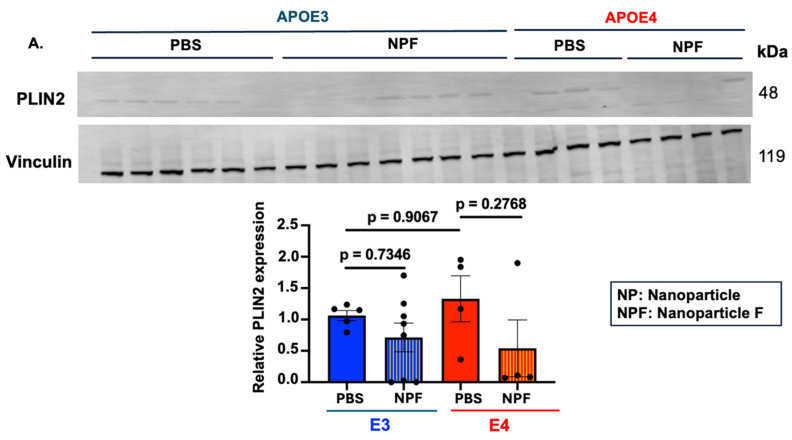

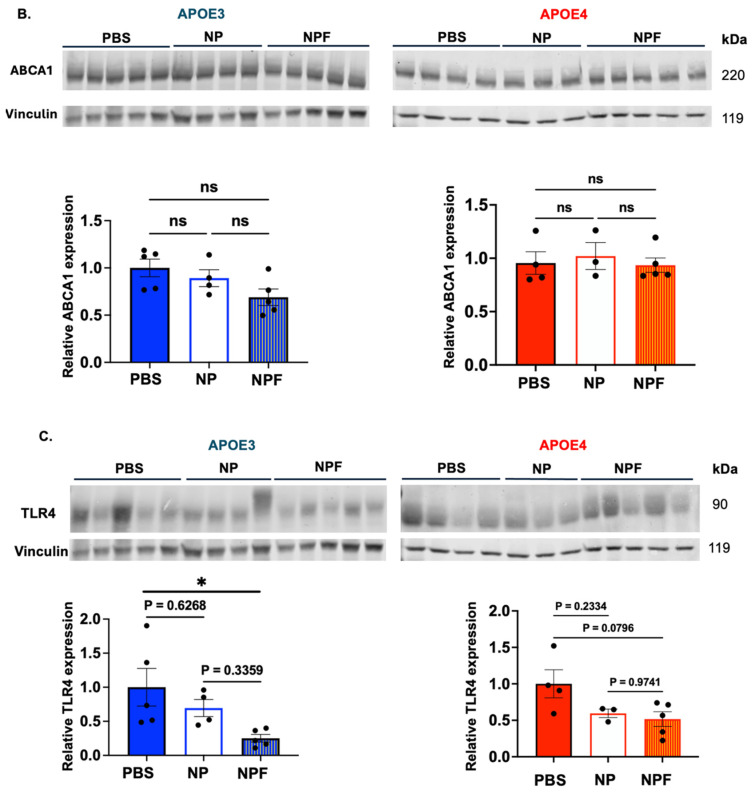

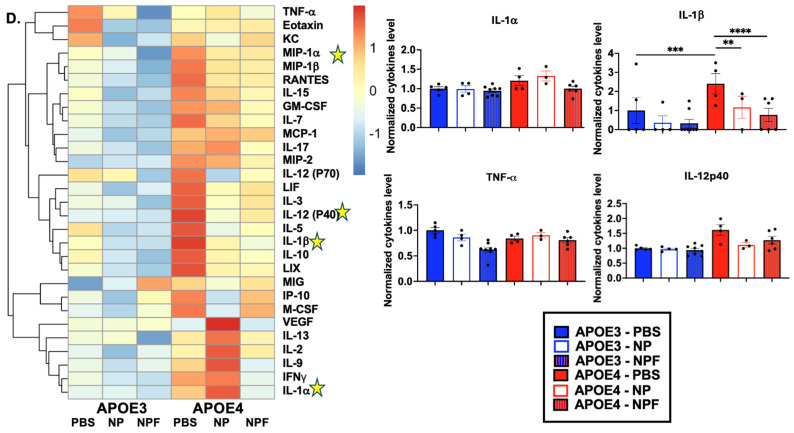
Two weeks of daily alternate IV and RO injection of nanoparticle F12511 at 46 mg/kg significantly alter the inflammatory profile and lipid droplet markers in both *APOE3* and *APOE4* forebrain at 16–20 M-old. (**A**) Representative Western blot and quantitative analysis of PLIN2 protein expression. (**B**) Representative Western blot and quantitative analysis of ABCA1 protein expression (**C**) Representative Western blot and quantitative analysis of relative TLR4 protein expression in 16–20 M-old, injected forebrain homogenate. (**D**) Heatmap visualizing average cytokines readings from each treatment group with Z-score transformation (left panel). Detectable Alzheimer’s-related cytokines and cytokines are significantly altered by drug treatment from MILLIPLEX xMAP analysis (highlighted by yellow stars) were plotted individually (right panels). The value of APOE3 mice treated with PBS was normalized to 1. Data are expressed as mean ± SEM. * *p* < 0.05, ** *p* < 0.01, *** *p* < 0.001, **** *p* < 0.0001.

To test whether NPF treatment reduces neutral lipid droplets in vivo, we monitored perilipin-2 (PLIN2) protein content in total forebrain lysate (Figure 5A). Previously, a decrease in PLIN2 signal was shown to be associated with reduced lipid droplet content [10,55]. As expected, and similar to our data in *APOE4* primary microglia, PLIN2 protein content in the forebrain of the nanoparticle F12511-treated group exhibited a decreasing trend compared to the PBS-treated group in both *APOE3* and *APOE4* mice, suggesting a reduction in neutral lipid droplet content in the brains of these mice. In principle, reducing brain CE-rich lipid droplets can release free cholesterol to participate in critical cellular processes [56]. This result agrees with previously published data by Valencia-Olvera et al., using a different ACAT inhibitor (avasimibe) and administration through a different route in *APOE4*/5xFAD mice [29].

We also determined the effect of NPF on ABCA1 protein content in vivo (Figure 5B). Our results demonstrated that in aging *APOE3* and *APOE4* mice, the nanoparticle and nanoparticle F12511 did not affect ABCA1 protein content. These data agree with our study in cell culture with *APOE4* microglia and astrocytes, which showed that blocking ACAT1 on ABCA1 protein expression levels may be cell-type specific (Figure 1C–F). In vivo, while F12511 increases ABCA1 protein content in *APOE4* microglia, it might not be able to induce ABCA1 protein expression in *APOE4* astrocytes and astrocytes counts for the majority of cells in the brain.

We observed a significant decrease in TLR4 protein content in aging APOE3 mice and a trending reduction in TLR4 protein content in aging *APOE4* mice (Figure 5C). This finding agrees with previous data from Li et al. In cell culture [28]. Li et al. showed that in LPS-induced N9 microglial cells, acute treatment (~4 h) with the ACAT1 inhibitor K604 modulates the fate of TLR4 and increases TLR4 endocytosis rates to the lysosome, while chronic treatment of the ACAT inhibitor (48 h) results in TLR4 protein degradation in the lysosomes [28]. Our result in Figure 5C suggested that a 2-week daily injection is sufficient to decrease TLR4 protein content in both aging *APOE3* and *APOE4* forebrains.

Finally, to determine whether the reduction of TLR4 protein content and the decrease in neutral lipid droplets correlate with an inflammatory profile in the brains of *E3/E4* mice, we analyzed aged 16–20 M-old forebrain tissue cytokines with MILLIPLEX xMAP technology in a similar manner to the analysis of the 9 M-old mice samples reported in Figure 4. Results from this screen are reported in Figure 5D: left panel comparing average cytokine levels between different treatment groups. As expected, 26 out of 31 screened cytokines were elevated in *APOE4* mice compared to *APOE3* mice, which suggests a more proinflammatory phenotype in *APOE4* (first and fourth column from the left, Figure 5D, left panel). This result is consistent with previous literature [10,11,31]. Treatment with nanoparticle F12511 results in a decreasing trend in the majority of proinflammatory cytokines in both *APOE3* and *APOE4* mice compared to PBS- and nanoparticle-alone-treated groups (Figure 5D, left panel). Treatment with nanoparticle alone (without F12511; NP) decreased certain cytokines levels compared to PBS-injected samples. Treatment with NPF further decreased certain proinflammatory cytokines, such as TNF-α in *APOE3* and IL-1β in *APOE3* and *APOE4*, suggesting that there might be a cooperative effect between nanoparticles with PC-NP and F12511 (Figure 5D, right panel). We plotted individual cytokines related to AD and neuroinflammation, such as IL-1α, IL-1β, TNF-α, and IL-12p40, to further assess their inflammatory profiles and observed decreasing trends of these cytokines in *APOE4* mice treated with NPF when compared to PBS treated or NP treated mice (Figure 5D, right panel). Specifically, the result shows that, out of all cytokines, NPF treatment significantly decreases IL-1β in *APOE4* mice. This observation is consistent with our previous data in N9 microglia cell culture Li et al. [28] and *APOE4* primary microglia in Figure 5C. Since IL-1β is released downstream of TLR4 activation, a decrease in TLR4 content is expected to result in lower levels of IL-1β secretion. Our data in Figure 5 support the conclusion that NPF decreases TLR4 protein content and lipid droplets and reduces proinflammatory cytokines in both aged *APOE3* and *APOE4* mice brains.

Our results show that, when inhibiting ACAT1 in vivo with nanoparticle F12511, a more pronounced effect is observed in aging mice compared to results obtained in adult mice (Figure 4B and Figure 5D). This result could be explained as follows: (1) The BBB is leaky in aging mice, allowing more drugs to enter the brain to carry out its therapeutic effect. (2) *APOE4*’s pathological phenotype is more significant with age, leading to a more measurable difference between the treated and the control groups. Previously, we demonstrated that in 3xTg mice, NPF treatment also resulted in a more notable effect in suppressing neuroinflammation in aging mice (16–20 M-old) compared to adult mice [30]. Our data in Figure 5D, left panel, showed that in aging mice at 16–20 M-old of age, *APOE4* mice exhibit more inflammation in the brain compared to its APOE3 counterparts, while this difference is more subtle in adult mice at 9 M-old of age; this result is consistent with previously reported data by Lee et al. [31].

Interestingly, we noticed that in aging mice, NP exhibited mild suppressive effects on TLR4 and IL-1β expression levels (Figure 5A and Figure 5C, right panel, respectively). We had previously made a similar observation in aging (16–20 months) 3xTg mice: nanoparticles with PC (NP) but without F12511 had a reducing effect on human hyperphosphorylated Tau, as well as a reducing effect on proinflammatory cytokine levels in selective cytokine markers, such as Eotaxin and TNF-α [30]. These observations led us to speculate that PC and F12511 encapsulated in DSPE-PEG_2000_ liposome may work in an additive manner, perhaps to strengthen lipid raft function, as discussed in [30], to suppress neuroinflammation. PC was previously demonstrated to exhibit anti-inflammatory properties in an LPS-induced systemic inflammation model [57]. Additionally, PC can alter lipid raft size, which serves as a signaling platform for TLR4 [58]. Since NP also contains DSPE-PEG_2000_, we cannot currently rule out the possibility that the effect of NP that we observed is mainly caused by the DSPEG-PEG_2000_ liposome. However, DSPE-PEG was reported to alter immune signaling via modulating TLR4 lipid raft signaling and augmenting proinflammatory responses [59]. The effect of DSPE-PEG in immune signaling is opposite to what we have observed with NP, which contains DSPE-PEG_2000_ and PC. Currently, it is unclear which cell type(s) are responsible for the in vivo effects of NP. We speculate that the vascular endothelial cells may be one of the primary targets of NP, as they are directly exposed to nanoparticle-containing PC upon NP injection into the bloodstream. Once the BBB becomes leaky, the PC in NP may interact with other cell types, such as pericytes, astrocytes, microglia, neurons, and oligodendrocytes. Future studies are needed to elucidate the in vivo effects of PC.

## 3. Discussion

In this study, we aimed to investigate the effect of ACAT1 inhibition in *APOE4* cell and mouse models. We showed that, in vitro, ACAT1 inhibition in *APOE4* primary microglia decreases CE-rich lipid droplets, upregulates ABCA1 protein levels, and dampens NFκB-mediated activation in a TLR4-dependent manner (Figure 1 and Figure 2). We also demonstrated that ACAT1 inhibition does not affect ABCA1 protein levels in an *APOE4* astrocytic cell line (Figure 1). We then tested the effect of inhibiting ACAT1 in vivo by using the lipid nanoparticle system containing DSPE-PEG2000, the ACAT1 inhibitor F12511, and PC on female adult (9 M of age) and aging (16–20 M of age) mice (Figure 3). Our results showed that in aging mice, NPF decreases TLR4 protein levels, reduces lipid droplet formation, and dampens proinflammatory cytokine secretion; the effects of NPF in the same mice strains in adulthood are less pronounced (Figure 4 and Figure 5).

**Figure 6:**
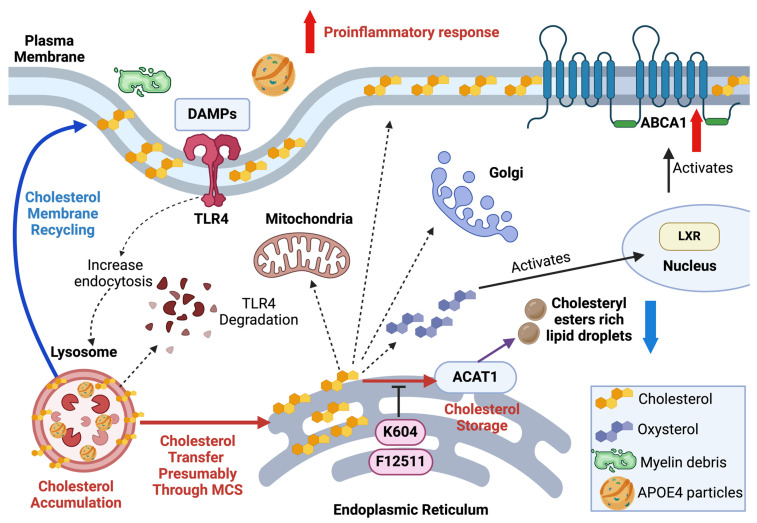
A working model to account for the effects of ACAT1 blockade in *APOE4* aging microglia. In aging and *APOE4*, myelin debris and disease-associated molecular patterns (DAMPs), including dead cell debris, are phagocytosed by resident microglia. *APOE4*-associated phenotypes are highlighted using red or blue arrows with red captions (increased activity) and blue captions (decreased activity). Cholesterol derived from myelin debris, APOE4 protein particles, or dead cells’ cholesterol-rich membranes enters microglial cells, increasing the cholesterol supply to ACAT1 at the ER. This activates ACAT1 through sterol-dependent allosteric control [62,63], leading to the conversion of cholesterol into CE (red arrow). When ACAT1 is inhibited (e.g., by using K604 or F12511), CE content decreases. Cholesterol diverted from the ACAT1 substrate pool can be converted into oxysterols, downregulating cholesterol biosynthesis by suppressing HMG-CoA reductase [27]. Additionally, this diversion upregulates ABCA1 gene expression for cholesterol efflux via an LXR-dependent pathway [32] (solid black arrows). Blocking ACAT1 also redirects cholesterol from the ACAT1 subdomain in the ER to the plasma membrane, promoting cholesterol efflux via ABCA1 and to other subcellular organelles, such as the Golgi and mitochondria. These cholesterol transfer steps likely occur through multiple membrane contact sites (MCS) between these organelles and the ACAT1 subdomain(s) in the ER [56,64,65]. APOE4 protein disrupts endo/lysosome function [8,10,11,41,60,66]. As endo/lysosomes malfunction, the process of membrane cholesterol recycling from endo/lysosomes back to the plasma membrane is impaired (blue arrow, blue caption). This results in defective intracellular cholesterol trafficking in a cell-type-dependent manner [7,8,9], leading to cholesterol accumulation in the lysosomes and increased proinflammatory responses. The accumulation of cholesterol in endo/lysosomes may also lead to an increase in ER cholesterol. Cholesterol transfer may occur through membrane contact sites (MCS) between endo/lysosomes and the ER (red arrow, red caption). Cholesterol in the ER then moves to the subdomain where ACAT1 resides, where it is esterified by ACAT1, resulting in the accumulation of CE-rich lipid droplets. ACAT1 blockade activates cholesterol transfer steps (shown as --------->) (dashed black arrows), which are not affected by APOE4 protein defects. ACAT1 inhibition may also influence cholesterol content and function by increasing endocytosis of membranes rich in TLR4 to the lysosome, promoting TLR4 degradation in the lipid raft microdomain that is enriched in TLR4 (Reviewed in [46]). Inhibiting ACAT1 activity leads to TLR4 degradation in lysosomes and dampens the proinflammatory response in these cells (dashed black arrows) [28]. The role of PC in the F12511 nanoparticle (Figure 8 in [30] and Figure 5A–D), may act within the lipid raft domain and work in concert with the ACAT1 inhibitor F12511. Created with BioRender.

In the mouse brain, APOE4 protein is defective in intracellular cholesterol trafficking, resulting in inefficient utilization of free cholesterol, which leads to cholesterol accumulation in the ER and subsequent CE formation in a cell-type-specific manner [8,9,11]. The exact mechanisms by which APOE4 protein causes these events remain poorly understood. Interestingly, a general mechanism has been proposed to explain APOE4 protein’s action: in various cell types, APOE4 alters the pH of endosomes and disrupts endosomal membrane recycling [60]. In addition, APOE4 protein disrupts lipid rafts and interferes with critical signaling events at the cellular membrane [61]. Such disruptions could lead to intracellular cholesterol trafficking defects observed in *APOE4* cells. Based on this and other information, we have developed a working model to explain the effects of ACAT1 blockade in *APOE4*-aging microglia, as shown in Figure 6. The details of this hypothesis are provided in the legend of Figure 6.

To summarize the hypothesis: *APOE4* disrupts endo/lysosomal membrane trafficking and recycling, damages lipid raft function, and disturbs cholesterol homeostasis. At the same time, ACAT1 blockade facilitates cholesterol transfer among various membrane organelles to meet the cholesterol demands of membranes, enhances lipid raft function, and supports cholesterol homeostasis. At the molecular level, ACAT1 inhibition operates independently of APOE protein and can bypass the cholesterol trafficking jam caused by APOE4 protein. The validity of this working model will need to be tested in future studies.

CE and TAG are the two main components of lipid droplets. Other studies have focused on studying TAG accumulation in *APOE4* cells [12,67] and reported that inhibition of TAG biosynthesis can control inflammation as well as suppress disease-associated gene expression in human *APOE4* iPSC-derived microglia [67]. Studies focusing on removing CE droplets and how that may benefit *APOE4* carriers have been rare. However, studies on removing CE droplets and how this may benefit *APOE4* carriers have been rare. Haney et al. showed that inhibiting ACSL1, a protein upstream of ACAT1 responsible for CE synthesis, and DGAT, which is responsible for TAG synthesis, in *APOE4* mice reduces disease pathology and ameliorates neuroinflammation [12]. Conceptually, using an ACSL1 inhibitor to reduce lipid droplets as a treatment for neurodegenerative diseases may be effective; however, its safety profile is a concern, partly because ACSL1 is also responsible for the bulk synthesis of phospholipids, which are essential components of all cell membranes [68]. Victor et al. demonstrated that in *APOE4* iPSC-derived microglia, ACSL1 inhibition by Triacsin C causes cytotoxicity [69].

A different approach by Blanchard et al. [9] removed CE lipid droplets in *APOE4* mice using cyclodextrin injection. Cyclodextrin is a non-specific cholesterol-sequestering agent that helps remove cellular cholesterol. This work supports the concept that *APOE4* expression leads to cholesterol trafficking defects. However, the safety profile of cyclodextrin is also a concern, as high concentrations of cyclodextrin have been shown to cause deafness in rodents [70]. Thus, an alternative approach to tackle lipid droplet accumulation in the *APOE4* model [69] may be warranted Our current work shows that removing CE is beneficial in the *APOE4* mouse model, which can be accomplished by specifically inhibiting ACAT1 using F12511. The FDA approved the safety of F12511 [71]. A separate study by Valencia-Olvera et al. [29] used another FDA-approved ACAT inhibitor, CI-1011, in the *APOE4*/5xFAD model and showed that CI-1011 dampens neuroinflammation in these mice. This work supports the use of ACAT inhibitors to treat Alzheimer’s disease (AD). However, it does not guarantee that ACAT1 inhibition will benefit the *APOE4* disease phenotype in the absence of an AD background (5xFAD).

Our current findings fill this gap in knowledge, demonstrating that in the absence of Aβ, ACAT1 inhibition is still beneficial in *APOE4* mice, particularly in suppressing neuroinflammation. We used *APOE4* primary microglia as a cell model to study the mechanism of ACAT1 inhibition and linked its effects to two membrane receptors at the plasma membrane: TLR4 and ABCA1. This mechanism may also apply to other cell types in the brain, including those at the blood-brain barrier (e.g., vascular endothelial cells, pericytes, etc.), which remains an open area of investigation. One shortcoming of our study is that due to the limited availability of aged mouse brain tissues, we were not able to conduct immunohistological staining, RNA-seq, and lipidomics experiments to further validate our Western blot and Luminex assay results. In the future, it is necessary to perform these experiments to strengthen our initial observation, as reported in this study.

Overall, our study suggests that ACAT1 inhibition is a novel strategy to ameliorate CE-rich droplets and dampen the pro-inflammatory response in the aging *APOE4* mouse model. Future studies should focus on how ACAT1 inhibition affects the *APOE4* phenotype in a cell-type-specific manner in vivo and validate these findings in human iPSC models.

## 4. Materials and Methods

### 4.1. Ethical Handling of Animals

All mouse experiments were conducted in accordance with ethical guidelines and approved by the Institutional Animal Care and Use Committee (IACUC) at Dartmouth College under protocol #00002020, approval date: 08/13/2024. *APOE3* and *APOE4* target replacement (*APOE3-TR* and *APOE4-TR*) mice on a C57B6/J background were procured from Taconic (La Jolla, CA, USA). Female mice aged 9 months and 16–20 months were used for in vivo studies, while primary microglia were isolated from mixed-gender P0-3 pups.

For in vivo studies, female *APOE3*-TR and *APOE4*-TR mice within the indicated age range were treated daily for 13–14 days by alternating between intravenous injections via the tail vein and retro-orbital venous injections at a dose of 46 mg/kg nanoparticle F12511. Mice were then perfused with 30 mL of ice-cold PBS (Millipore Sigma, St. Louis, MO, USA) to remove blood contaminants. Forebrain tissues were collected and snap-frozen on dry ice. Tissues were stored at −80 °C until further processing.

### 4.2. Cell Culture

Primary microglia were isolated from P0-P3 pups according to procedures previously described [72]. Briefly, brains were isolated, and meninges were removed. The brains were homogenized by mechanical pipetting and trypsinization and then plated on poly-L-lysine-coated flasks. Cells were cultured in DMEM with 10% heat-inactivated fetal bovine serum (FBS) and 1% penicillin/streptomycin (P/S), all from Gibco (ThermoFisher, Waltham, MA, USA), overnight. The next day, cells were rinsed with PBS and cultured in DMEM supplemented with 10% FBS, 1% P/S, and 1% L929 conditioned medium (growth factors) for 10 to 14 days. Primary microglia were harvested by shaking at 225 rpm at 37 °C for 30 min and plated overnight in DMEM supplemented with 10% FBS and 1% P/S. Prior to treatment, cells were switched to DMEM free of serum and antibiotics. Immortalized mouse astrocytes expressing human *APOE3* and *APOE4* were a gift from Dr. David Holtzman, grown in DMEM/F12 supplemented with 10% FBS, 1% P/S (ThermoFisher, Waltham, MA, USA). All cells were maintained at 37 °C with 5% CO₂ in a humidified incubator.

### 4.3. Nile Red Staining in Live Cells and Images Analysis

According to previously described procedures, Nile Red was used to monitor lipid droplets in live cells [37]. Briefly, primary microglia were plated on 35 mm dishes pre-coated with poly-L-lysine at 2 × 10⁶ cells per plate overnight. Treatment was performed in serum-free DMEM, and cells were rinsed three times with HBSS (Corning, NY, USA). Cells were treated with 100 ng/mL Nile Red and incubated for 10 min at 37 °C, 5% CO₂, protected from light. Cells were rinsed in HBSS and imaged in DMEM serum-free, with no phenol red (Gibco by ThermoFisher, Waltham, MA, USA), on the confocal fluorescence microscope. Data were collected and analyzed for image analysis using Fiji-ImageJ software version 2.1.0/1.53c, following the same pipeline described in [73].

### 4.4. Whole Cell Protein Isolation, Tissue Homogenization, and Western Blot Analyses

To obtain whole cell lysates, cells were harvested in RIPA buffer containing a protease inhibitor cocktail (Millipore Sigma, St. Louis, MO, USA) and incubated at 4 °C for 30 min. For NFκB Western blot, cells were lysed directly in sample buffers containing DTT, supplemented with protease inhibitor cocktails and PHOsSTOP phosphatase inhibitors (Roche, Indianapolis, IN, USA). RIPA cell lysates were then centrifuged, and the protein concentration of the supernatant was determined by Lowry protein assay.

To prepare brain homogenates, half of the frozen, perfused forebrains from female *APOE3-TR* and *APOE4-TR* mice within the indicated age ranges were homogenized with stainless steel beads in the Bullet Blender at 4 °C in sucrose-based buffer, supplemented with a protease inhibitor cocktail as previously described in [27,30]. Brain homogenates were centrifuged at 12,000× *g* for 15 min, and supernatants were collected for Western blot and Luminex analysis.

The lysates or homogenates were run on (16 cm × 17.5 cm) 6% SDS-PAGE gels (50 μg of total protein) or precast mini 4–20% Novex Tris-Glycine gels (15 μg of total protein) (ThermoFisher, Waltham, MA, USA) and transferred to a 0.45 μm nitrocellulose membrane for 4 h at 300 mA, or a 0.2 μm PVDF membrane for 1 h at 200 mA. Membranes were blocked in 5% milk in 1× TBS or 5% BSA in 1× TBS for phosphorylated protein detection. For primary antibody detection, the membranes were incubated overnight with anti-ABCA1 (Novus, Littleton, CO, USA NB400-105), anti-PLIN2 (Proteintech Rosemont, IL, USA 15294-1-AP), anti-TLR4 (Santa Cruz Dallas, TX, USA sc-293072), IκBα (CST, Danvers, MA, USA #4814), phospho-IκBα Ser32 (CST, Danvers, MA, USA, 14D4), phospho-NFκB p65 Ser536 (CST, Danvers, MA, USA, 93H1), NFκB p65 (CST, Danvers, MA, USA, L8F6), and anti-Vinculin (Millipore St. Louis, MO, USA 05-386) as a protein loading control. Blots were then washed and incubated with secondary antibodies IRDye^®^ 680RD goat anti-mouse (P/N 926-68070) and IRDye^®^ 800 W goat anti-rabbit (P/N 926-32211) from Li-Cor (Lincoln, NE, USA). Western blot images were captured on the Li-Cor Odyssey CLx and analyzed on Li-Cor Image Studio. Treatment schemes for individual experiments are shown in the Supplementary Materials, Figures S1 and S2.

### 4.5. Preparation of Nanoparticle and Nanoparticle F12511

Nanoparticles F12511 were prepared according to procedures previously described [30,49]. Briefly, DSPE-PEG2000 (Laysan Bio, Arab, AL, USA) in EtOH, L-α-Phosphatidylcholine (from egg yolk) (MilliporeSigma, St. Louis, MO, USA) in chloroform, and F12511 (Wuxi AppTec, Wuxi, China) in EtOH were combined, vigorously vortexed, and lyophilized overnight at −40 °C, with the vacuum set at 133 × 10^−3^ mBar. Sterile PBS was then added to the lyophilized mixture so that the final concentration of the nanoparticle F12511 solution contained 30 mM DSPE-PEG2000, 6 mM PC, and 12 mM F12511. Mixtures were sonicated at 4 °C for 2–4 cycles, each lasting 20 min. The sonicated solutions were spun at 12,000 rpm for 5 min, and supernatants were collected for animal injections. The resulting solution was protected from light throughout the entire process. For nanoparticles without the F12511 formulation, the solution was prepared following the same procedure, except F12511 was omitted.

### 4.6. Luminex Analysis

Luminex analysis was performed using the procedure described previously [30]. Cytokines from the mouse brain homogenates prepared in Section 4.5 were measured using Millipore mouse cytokine multiplex kits—MILLIPLEX xMAP (EMD Millipore Corporation, Billerica, MA, USA). Calibration curves from the recombinant cytokine standards were prepared by following threefold dilution steps in the same matrix as the samples. High- and low-quality control samples with a known concentration provided by the manufacturer were used to validate the standard curve calculation. The standards and quality control samples were measured in triplicate, while the samples were measured once, and blank values were subtracted from all readings to ensure accurate measurement. All assays were carried out directly in a 96-well filtration plate (Millipore, Billerica, MA, USA) at room temperature and protected from light. Briefly, each well was pre-wet with 100 µL of PBS containing 1% BSA. Then, the beads, along with the standard sample, quality control samples, or blanks, were added to a final volume of 100 µL. The plate was incubated at room temperature for 30 min with continuous shaking. The beads were washed three times with 100 µL of PBS containing 1% BSA and 0.05% Tween 20. A cocktail of biotinylated antibodies (50 µL/well) was added to the beads for a further 30 min of incubation with continuous shaking. The beads were washed three times, and then streptavidin-PE was added for 10 min. The beads were again washed three times and resuspended in 125 µL of PBS containing 1% BSA and 0.05% Tween 20. The fluorescence intensity of the beads was measured using the Bio-Plex array reader 200 from Bio-Rad (Hercules, CA, USA). Bio-Plex Manager software Version 6.2 with five-parameter curve fitting was used for data analysis.

### 4.7. F12511

F12511 was custom synthesized by WuXi AppTec in China based on the published method [74], with verified stereospecificity and 98% purity by Mass spectrometry and NMR. F12511 is a potent ACAT inhibitor that strongly inhibits ACAT1 (K_i_ = 0.039 μmol/L) and ACAT2 (K_i_ = 0.110 μmol/L). F12511 is a derivative of fatty acid anilide. F12511 is a noncompetitive inhibitor of ACAT.

### 4.8. Statistical Analysis

All statistical analysis was performed using Prism10 software (GraphPad, La Jolla, CA, USA). A one-way ANOVA test with Sidak multiple comparisons was used to analyze data between treatment groups. A two-way ANOVA with Turkey multiple comparison test was used for Luminex analysis. Error bars indicate SEM. * *p* < 0.05; ** *p* < 0.01; *** *p* < 0.001; ****p < 0.0001.

## Data Availability

Dartmouth College has filed a U.S. Provisional Application entitled “Method for Attenuating Neuroinflammation, Amyloidopathy and Tauopathy”, application number US20220257544A1. The authors declare that the relevant data are included in the article.

## Supplementary Materials

The following supporting information can be downloaded at: https://www.mdpi.com/article/10.3390/ijms252413690/s1.
