# Supplementary material for: Inhibiting the Cholesterol Storage Enzyme ACAT1/SOAT1 in Aging Apolipoprotein E4 Mice Alters Their Brains’ Inflammatory Profiles
Source: Int J Mol Sci. 2024 Dec 21;25(24):13690. doi: 10.3390/ijms252413690 (PMC11727783; doi:10.3390/ijms252413690)
Supplement: Supplementary file 1 [file ijms-25-13690-s001.zip › ijms-3366332-supplementary.pdf]

IJMS 3366332 – Supplementary Figures

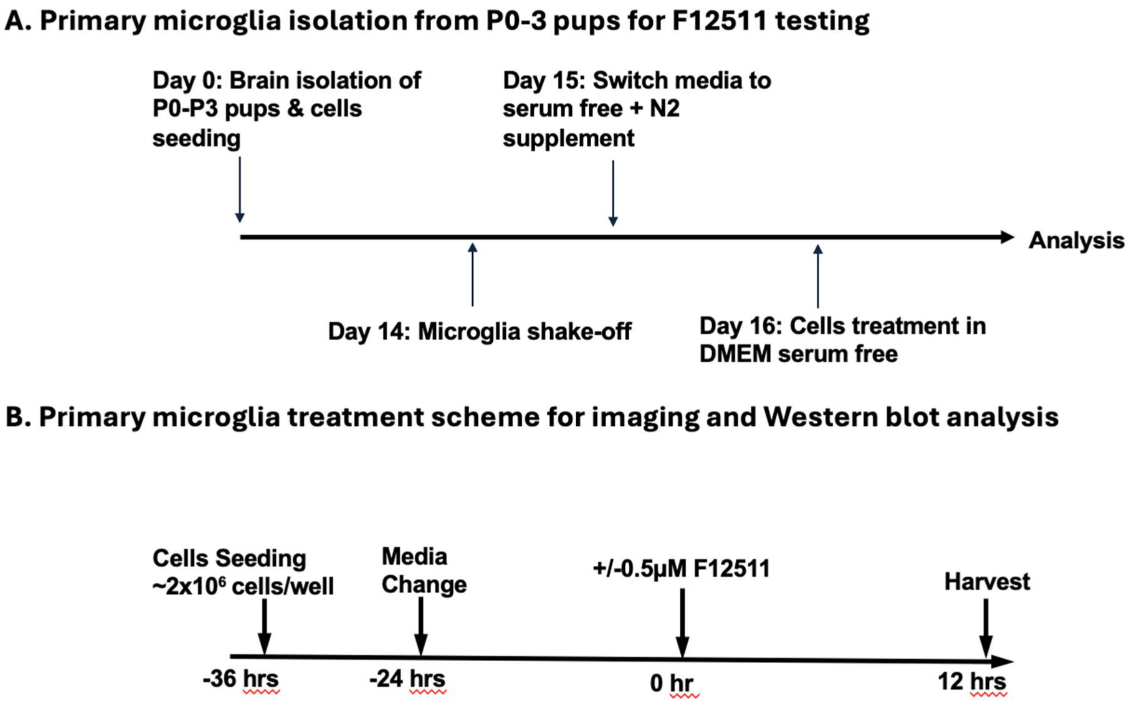

Figure S1. In vitro F12511 treatment APOE4 primary microglia and immortalized astrocytes. (A) Experimental timeline for primary microglia isolation from P0-3 pups. **(B)** Primary microglia treatment scheme for imaging and Western blot analysis in serum-free media.

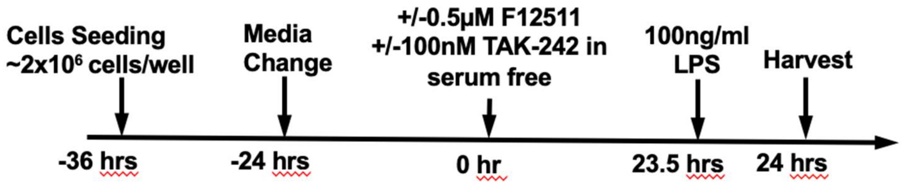

Figure S2. Pharmaceutical inhibition of ACAT1 by F12511 in LPS-induced APOE4 primary microglia. Treatment scheme of APOE4 primary microglia. LPS (inflammatory activator) is used to activate primary microglia after incubation with F12511 (ACAT inhibitor) with or without TAK-242 (TLR4 inhibitor).
